# Multiple Low Frequency Ultrasound Enhances Bactericidal Activity of Vancomycin against Methicillin-Resistant* Staphylococcus aureus* Biofilms

**DOI:** 10.1155/2018/6023101

**Published:** 2018-09-30

**Authors:** Jin Wang, Ke Wen, Xu Liu, Chun-xiao Weng, Rui Wang, Yun Cai

**Affiliations:** ^1^Center of Medicine Clinical Research, Department of Pharmacy, PLA General Hospital, Beijing, China; ^2^Chiamery Medical Sciences Institute of Beijing, Beijing, China

## Abstract

Methicillin-resistant* Staphylococcus aureus *(MRSA) biofilm infections are difficult to treat due to the high antimicrobial resistance of biofilm. Therefore, new treatments are needed for more effective bacteria clearance. This study was to investigate whether low frequency ultrasound (LFU) can enhance the activity of antimicrobial agents against MRSA biofilm infection. Broth microdilution method was used to determine the minimum inhibitory concentration (MIC) of vancomycin (VAN), linezolid (LIN), and levofloxacin (LEV) against three clinical isolated strains, including one methicillin-susceptible* Staphylococcus aureus* (MSSA) strain and two MRSA strains. Effects of various influencing factors, such as antimicrobial agents, drug concentrations, ultrasonic intensity, and single (S-LFU, 5 or 15 min) or multiple ultrasound (M-LFU, 5 min every 8 h), on the inhibition of biofilms were investigated. The bactericidal effects of S-LFU or M-LFU on MRSA or MSSA biofilms were determined by colony counts. Right after ultrasound, synergistic effects were observed in groups of S-LFU combined with three antimicrobial agents against MSSA biofilm, but for MRSA biofilm, only S-LFU plus VAN had synergistic effect. At the time point of 24 h, M-LFU plus VAN treatment had synergistic bactericidal effect against MRSA and MSSA biofilms, and the synergy showed that VAN is concentration-dependent, but no synergistic effects were observed in all S-LFU combination groups. In conclusion, combination of M-LFU and antimicrobial agents had a better synergistic effect than S-LFU against MRSA or MSSA biofilm. LFU may be useful in treating biofilm infection in the future.

## 1. Introduction

Treatment of methicillin-resistant* Staphylococcus aureus *(MRSA) biofilm infections is a hot topic in the field of antimicrobial resistance and opinions are in constant flux [1]. In clinic, MRSA biofilm can grow on almost all implanted medical devices, including catheters, prosthetic heart valves, and cardiac pacemakers, which can lead to biofilm infections [2]. Biofilm associated infections are difficult to treat because the biofilm matrix and phenotypic characteristics of the bacteria in biofilms confer resistance to the host immune response and the action of antimicrobial drugs [3]. It is urgent to find alternative therapeutic strategies that allow the use of less antibacterial agents and, therefore, reduce bacterial resistance [4]. Combination therapy may overcome some of the drug limitations and provide more time for new drugs to be routinely administered [5]. However, combination therapy of licensed drugs often lacks clear clinical evidence of its benefit and may be associated with bad outcome [6]. Unorthodox combination of low frequency ultrasound (LFU) and antimicrobial agents may bridge the gap in current treatment against biofilm infections. As a noninvasive physical method, ultrasonic irradiation can be achieved under ambient conditions of pressure and temperature without any chemical compounds, making it one of the most attractive methods [7].

In this study, we investigated the synergy of LFU in combination with antimicrobial agents on MRSA and MSSA biofilms. The possible influencing factors, including ultrasonic time, intensity, frequency (S-LFU or M-LFU), antimicrobial agents (VAN, LEV, or LIN), antibiotic concentration, and types of biofilms (MRSA or MSSA biofilm), were studied* in vitro*.

## 2. Materials and Methods

### 2.1. Strains, Agents, and Antimicrobial Susceptibility Test

Two MRSA strains numbered R1582 and 152799 were clinically isolated from People's Liberation Army General Hospital and were identified by the automated VUTEK 2 Compact System (BioMerieux, Marcy-l'Etoile, France) microbe analyzer.* Staphylococcus aureus *ATCC 29213, which was methicillin-sensitive, was used as the quality control strain. Susceptibility test of all antimicrobial agents was performed in duplicate by broth microdilution, according to CLSI guide lines. Briefly, 96-well plates were set up with LIN (Pfizer, Madison, USA),VAN or LEV (National Institute for the Control of Pharmaceutical and Biological Products, Beijing, China) from 0.0075 to 256 *μ*g/mL. Strains were, respectively, grown on Mueller-Hinton agar (BD Difco, Franklin Lakes, NJ, USA), and representative colonies were picked up and suspended in Mueller-Hinton broth, MHB (BD Difco, Franklin Lakes, NJ, USA). For each strain, a 100 *μ*L volume of resultant bacterial suspension (1×10^5^ colony forming units) was loaded into each well of a 96-well plate and then incubated at 37°C [8].

### 2.2. Cultivation of Biofilm

The biofilm was cultivated according to procedure described in previous study. Briefly, each strain was cultivated on catheter disks (diameter=0.5 cm) in 24-well plates. In each well, three disks, 1 mL MHB and 100 *μ*L bacterial suspension (1.5×10^8^ cfu/mL), were added. The 24-well plates were incubated at 37°C for 2 days. MHB was renewed every day [9].

### 2.3. LFU Apparatus

**Figure 1:**
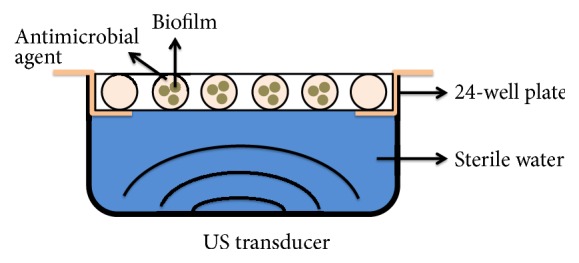
Diagram depicting using LFU and antimicrobial agents for the treatment of MRSA or MSSA biofilms. Three catheter disks with biofilms were placed into each well of a 24-well plate that contained 1 mL of vancomycin, linezolid, or levofloxacin solution. Sterile medium was added to the wells around the edge of the 24-well plate, serving as a negative control. LFU was transmitted through the bottom of the plate via sterile water.

LFU apparatus was provided by Beijing Nava Medical Technology. S-LFU was operated at 40 kHz, with continuous irradiation at intensity of 92.36, 138.54, and 184.72 mW/cm^2^ for 5 or 15 min and M-LFU was operated at 40 kHz, 92.36 mW/cm^2^, and 5 min every 8 h (q8h) on biofilms [9]. The experimental setup is shown in Figure 1. In each well of 24-well plate, three disks with biofilm and 1 mL MHB with or without agents were added. To avoid the effect of the holder, antimicrobial agent solution without a disk was added to the wells around the edge of the 24-well plate. The ultrasonic transducer was placed 7 cm below the wells, and LFU was transmitted through the bottom of the 24-well plate via sterile water in a LFU bath [10]. There was no difference in water temperature before and after ultrasound treatment.

### 2.4. Measurements of the Bactericidal Activity of S-LFU and M-LFU in combination with VAN, LEV, or LIN

We used 92.36 mW/cm^2^ power intensity and adjusted irradiation time to 5 or 15 min for S-LFU and 5 min every 8h for M-LFU. The biofilm disks of three bacterial strains were treated with 4×MIC VAN, LEV, and LIN with or without LFU [11]. After S-LFU or M-LFU processing, the 24-well plates were incubated at 37°C for 24 hours. The disks were taken out either right after ultrasound or 24 hours later and washed with saline three times to remove planktonic bacteria. The adherent bacteria were collected from the disks using an ultrasonic cleaning bath for 10 min. The bacterial solution was vigorously mixed and plated on agar plates as 10-fold serial dilutions and cultured for 16~24 h. The colony numbers between 30 and 300 per plate were considered as good results. Each treatment had six catheters. Bacteria counts were repeated three times.

### 2.5. Measurement of M-LFU in combination with Different Concentrations of VAN

M-LFU was set as the power intensity of 92.36 mW/cm^2^, 5 min every 8 h. The biofilm disks of the three strains were treated with 1, 2, and 4×MIC VAN with or without M-LFU. At 24 hours following M-LFU, the viable bacteria counts were determined using the same method as described in measurements of bactericidal activity.

### 2.6. Statistics

Statistical analysis was performed with GraphPad Prism software (San Diego, CA, USA). Data were presented as mean ± SD. Comparisons were carried out using one-way analysis of variance (ANOVA) followed by Tukey-Kramer's teat for post hoc analysis.* P*<0.05 was considered as statistically significant.

## 3. Results

### 3.1. MICs for Antimicrobial Agents

The MICs are summarized in Table 1. Two MRSA strains were susceptible to VAN and LIN, but resistant to LEV. ATCC 29213 was susceptible to all agents.

### 3.2. Activity of S-LFU plus Antimicrobial Agents against MRSA or MSSA Biofilms

**Figure 2:**
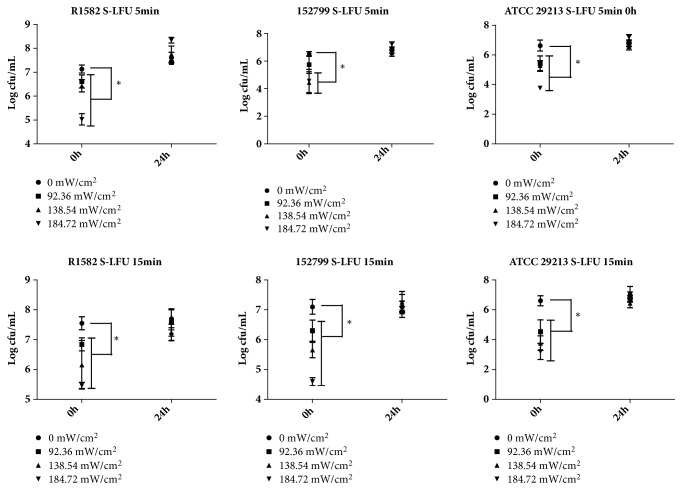
The effect of intensity and irradiation time on the bactericidal effect of S-LFU. Biofilms on disks were incubated at 37°C for 2 days. The biofilms of the three bacterial strains were treated with S-LFU with different intensity (92.36, 138.54, and 184.72 mW/cm^2^) and irradiation time (5 or 15 min). The viable bacterial counts in biofilms were determined right after ultrasound and at 24 hours following ultrasound. ^*∗*^*P*<0.05, as compared with the control group.

When the intensity of S-LFU (5 min or 15 min) was increased, viable bacterial counts in all groups were decreased right after ultrasound, but no differences were observed at 24 hours. The effect of S-LFU was more evident in MSSA (ATCC 29213) than MRSA (R1582 and 152799) biofilms (Figure 2).

**Figure 3:**
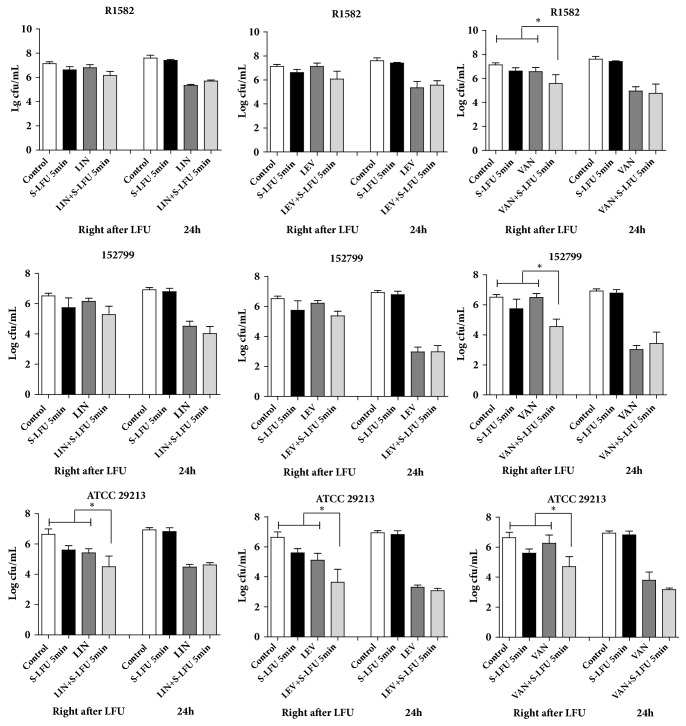
Synergistic effect of S-LFU and antimicrobial agents against MRSA or MSSA biofilms. S-LFU (92.36 mW/cm^2^, 5 min), in combination with antimicrobial agents at 4×MIC, was used to treat the biofilms. The viable bacterial counts in biofilms were determined right after ultrasound and at 24 hours. VAN, LIN, and LEV indicate vancomycin, linezolid, and levofloxacin, respectively. ^*∗*^*P*<0.05, as compared with the control, LFU, or antimicrobial agent treatment without LFU groups.

We treated the bacterial biofilm disks with S-LFU (irradiation 5 min) in combination with VAN, LEV, and LIN at 4×MIC. Viable bacterial counts in biofilms were determined by plate agar right after ultrasound and 24 hours later. Compared with antimicrobial agent without S-LFU, viable bacterial counts were significantly decreased in S-LFU plus VAN, LEV, or LIN in MSSA (ATCC 29213) biofilm, but only S-LFU plus VAN could decrease the bacterial counts in MRSA biofilms right after ultrasound. No synergistic effects were observed in S-LFU plus any antimicrobial agents in all biofilms at 24 hours (Figure 3).

### 3.3. The Effect of S-LFU or M-LFU on Synergistic Effect

**Figure 4:**
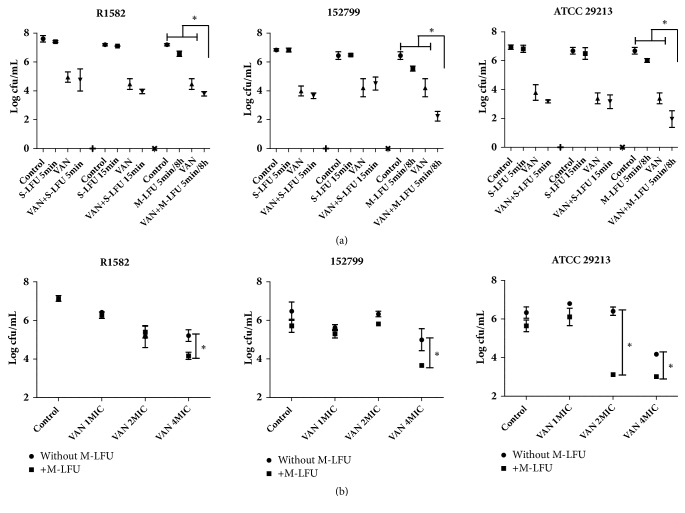
Synergistic effect of M-LFU and vancomycin against MRSA or MSSA biofilms. (a) Comparison of synergistic effects of S-LFU and M-LFU combined with vancomycin against MRSA or MSSA biofilms. S-LFU irradiation time was set to 5 and 15 min and M-LFU was set to5 min every 8 hours for 24 hours. With fixed intensity (92.36 mW/cm^2^), the synergistic effects of S-LFU and M-LFU in combination with 4×MIC vancomycin were investigated. The viable bacterial counts in biofilms were determined at 24 hours following ultrasound treatment. VAN refers to vancomycin. ^*∗*^*P*<0.05, as compared with control, vancomycin treatment without S-LFU or M-LFU group. (b) The effect of vancomycin concentration on the synergistic effect of vancomycin and ultrasound. With irradiation time adjusted to 5 min every 8 hours for 24 hours and fixed intensity (92.36 mW/cm^2^), M-LFU in combination with 1, 2, and 4×MIC vancomycin was used to treat the biofilms. The viable bacterial counts in biofilms were determined at 24 hours. VAN refers to vancomycin. ^*∗*^*P*<0.05, as compared with vancomycin treatment without M-LFU group.

Synergistic effects of S-LFU and M-LFU plus VAN were compared. For M-LFU plus VAN groups, the impact of the concentration of VAN was also investigated. At 24 hours, M-LFU plus VAN had synergistic effects in all biofilms, but the synergistic effect was not found in S-LFU combination groups (Figure 4(a)). We also determined the activity of M-LFU plus LEV and LIN and found that only M-LFU plus LEV showed synergy in 152799 biofilm (data not shown).

Viable bacterial counts in all biofilms were decreased as VAN concentration increased. For MRSA biofilms, synergistic effects were observed with M-LFU plus VAN at 4×MIC, but not with M-LFU plus VAN at 1 or 2×MIC when compared with agent alone without M-LFU. For MSSA biofilm, synergistic effects were observed with M-LFU plus VAN at 2 or 4×MIC, but not with M-LFU plus VAN at 1×MIC when compared with VAN without M-LFU (Figure 4(b)).

## 4. Discussion

A growing number of studies demonstrated synergistic effect of LFU in combination with antimicrobial agents against biofilm infections* in vitro *and* in vivo *[12–14]. In this study, combinations of S-LFU or M-LFU and antimicrobial agents were more effective than LFU alone against biofilms (Figures 2 and 3). That demonstrated the synergistic effect of LFU in combination with antimicrobial agents against MRSA or MSSA biofilm. The exact mechanism of synergy is unclear. Several studies suggested that LFU cavitation was mainly responsible for the synergistic antimicrobial effect [14–16]. Under LFU treatment, liquid medium could form microbubbles, which might act on biofilms and increase its permeability to antimicrobial agents or even kill bacteria in biofilm. Using laser confocal scanning microscope, Peterson et al. [17] observed that ultrasound created many holes in the extracellular matrix of bacterial biofilms and thus facilitated antimicrobial agents to enter biofilm. Meanwhile, with more oxygen and nutrition entering biofilms, the susceptibility of MRSA or MSSA to antimicrobial agents may be reversed [18]. Other studies indicated that synergy may also be associated with heating or other mechanisms [16, 19]. Overall, LFU possibly acts on the extracellular matrix of bacterial biofilm through multiple paths to facilitate the quick entrance of antimicrobial agents into biofilm. But for this study, heating most likely was not important since no differences in temperature were observed before and after LFU exposure.

Many factors affect the activity of LFU against biofilms. For example, the LFU parameters, including intensity, frequency, irradiation time, and duty cycle, are of significance [20]. Viable bacterial counts in biofilms were decreased right after ultrasound when increasing the S-LFU intensity. However, no differences were observed at 24 hours (Figure 2). It may prove the short-effect of LFU. Besides, viable bacteria counts were significantly decreased in biofilms after M-LFU plus VAN treatment, but not in S-LFU plus VAN treated biofilms (Figure 4(a)). It indicated that synergy could be enhanced by increasing the number of LFU times but not by prolonging LFU irradiation time within 24 hours. M-LFU may compensate for the short effect of LFU and led to a better effect.

In addition to LFU parameters, Ananta E [21] stated that the antimicrobial effect of ultrasound diverges in gram-negative and gram-positive bacteria due to the distinguishing feature of bacterial cell structure. Even for the same kind of bacteria, there are still some differences in the synergistic effects. Rediske AM [22] compared the antibacterial effects of aminoglycosides and tetracycline with or without LFU on three gram-positive bacteria strains. Synergy of LFU with tetracycline could be observed in* Enterobacter aerogenes *but not in* Serratia marcescens.* All S-LFU combination groups have synergy against MSSA biofilm, but only one group, S-LFU plus VAN, could decrease the bacterial counts against MRSA biofilms right after ultrasound (Figure 3). Those results indicated that MRSA biofilm-former strains may be more resistant to LFU treatment than MSSA biofilm. Numerous studies have indicated that MRSA and MSSA biofilms are different and this difference may affect the synergistic effects of LFU and antimicrobial agents. Biofilm formation is influenced by a variety of conditions and regulatory factors. There are some differences between MSSA and MRSA biofilms. MSSA strains form ica-dependent, polysaccharide intercellular adhesion-mediated biofilms. But MRSA strains formed biofilms are independent of polysaccharide intercellular adhesion. It requires surface proteins, such as the fibronectin binding proteins, Atl-mediated cell lysis, and eDNA, for colonization of surfaces and biofilm accumulation [2, 23]. The mechanism is unclear and needs further study.

Besides, antimicrobial agent is also a factor that affects the synergy between ultrasound and the antimicrobial agent. Liu B [11] found antibacterial effect of LFU and fluoroquinolones against* Escherichia coli *and this effect was influenced by fluoroquinolones concentration and temperature.* Enterobacter aerogenes* appeared to be more resistant to streptomycin than gentamycin or kanamycin when those drugs were combined with LFU [22]. Synergy of S-LFU or M-LFU with VAN was observed in MRSA and MSSA biofilms, but not with LIN and LEV (Figures 3 and 4). Thus, we proposed that the potent synergistic mechanism not only may be changing in biofilm permeability but is also influenced by antibiotics. Although LFU could enhance the permeability of biofilms, LIN and LEV may not entirely enter biofilms. The local drug concentrations were lower than MIC of the strains. However, VAN may penetrate the biofilm due to its unique mechanism. VAN was considered to be the last-resort antibiotic for the treatment of MRSA infections, but MRSA resistance to VAN has been reported too. MRSA may acquire more resistance to VAN in the near future [24, 25]. Therefore, it is urgent to discover new antibiotics or to devise new measurements that are effective against MRSA infections. LFU could improve the bactericidal effect of VAN and may slow the development of VAN resistance in MRSA. In addition, although VAN was a time-dependent antimicrobial agent, it showed a concentration-dependent profile against MRSA or MSSA biofilm in this study (Figure 4(b)).

Currently, only few clinical reports exist on testing the synergy of LFU and antibiotics against catheter-related biofilm infections in patients. Significant efforts are being exerted to develop noninvasive and effective treatments for biofilm infections. LFU, as an adjuvant tool, has been studied in chronic wound healing and offers relatively painless debridement and bacterial biofilm destruction. For the treatment of patients with infected prosthetic vascular grafts, Michele C performed an ultrasound debridement plus a partial graft removal or no removal instead of the traditional treatment that completes graft removal. They proved that ultrasound debridement was a valuable aid [26]. M-LFU may be applied in clinical practice and this study proved that M-LFU has distinct potential to facilitate antibiotics and obtain better effect than S-LFU. Although every 8 hours M-LFU is troublesome, the reasonable time of M-LFU will be investigated in the future.

In conclusion, we found that M-LFU is better than S-LFU to enhance the bactericidal effect of antimicrobial agents in MRSA and MSSA biofilms. Synergistic effects of M-LFU or S-LFU combined with VAN were observed in all biofilms and showed that VAN is concentration-dependent. The* in vitro* data presented here will support further investigations concerning the mechanism involved in the synergistic effect of LFU and VAN, as well as its applications* in vivo.*

## Figures and Tables

**Table 1:** MICs of antimicrobial agents against MRSA andMSSA strains.

Antimicrobial agents	MIC(*μ*g/mL)			MIC( interpretive criterion(*μ*g/mL)			Quality control standard (ATCC 29213)
	R1582	152799	ATCC 29213	Susceptible	Intermediate	Resistant	
VAN	1	2	0.5	⩽2	4-8	*⩾*16	0.06-0.5
LIN	1	2	2	⩽4	-	*⩾*8	1-4
LEV	32	32	0.5	⩽1	2	*⩾*4	0.5-2

MIC: minimum inhibitory concentration.

VAN:vancomycin; LIN:linezolid; LEV: levofloxacin.

MIC interpretive criterion and quality control standard (ATCC 29213) are based on Clinical and Laboratory Standards Institute guideline.

## Data Availability

The data used to support the findings of this study are included within the article.
